# Cellular functions and molecular mechanisms of ubiquitination in osteosarcoma

**DOI:** 10.3389/fonc.2022.1072701

**Published:** 2022-12-01

**Authors:** Jiaxun Song, Xiaofeng Yuan, Lianhua Piao, Jiawen Wang, Pu Wang, Ming Zhuang, Jie Liu, Zhiwei Liu

**Affiliations:** ^1^ The Third Affiliated Hospital of Soochow University, Changzhou, China; ^2^ Jiangsu University of Technology, Changzhou, China; ^3^ Changzhou Maternity and Child Health Care Hospital Affiliated to Nanjing Medical University, Changzhou, China

**Keywords:** Osteosarcoma, ubiquitination, deubiquitination, post-translation modification, cell signals

## Abstract

Although some advances have been made in the treatment of osteosarcoma in recent years, surgical resection remains the mainstream treatment. Initial and early diagnosis of osteosarcoma could be very difficult to achieve due to the insufficient sensitivity for the means of examination. The distal metastasis of osteosarcoma also predicts the poor prognosis of osteosarcoma. In order to solve this series of problems, people begin to discover a new method of diagnosing and treating osteosarcoma. Ubiquitination, as an emerging posttranslational modification, has been shown to be closely related to osteosarcoma in studies over the past decades. In general, this review describes the cellular functions and molecular mechanisms of ubiquitination during the development of osteosarcoma.

## 1 Osteosarcoma

In 1804, John Abernathy, a British surgeon, first proposed the concept of “sarcoma” (1). In 1805, French surgeon Alexis Boyer first used osteosarcoma to describe related types of disease. Boyer believed that osteosarcoma is a novel type of bone tumor that differs from other types of bone tumors (2). Osteosarcoma, a malignant aggressive tumor arising from the mesenchyme, accounts for 20% of all primary bone tumors and is characterized by the formation of immature osteoid tissue (3, 4). Osteosarcoma occurs in the metaphysis of long bones and higher rates are reported among male than female youth, with a male-to-female ratio close to 1.4:1 (5, 6). Patients usually present initially with local pain and limitation of movement at the lesion site and distal metastases in the late stage. The most metastatic organ is the lung (7). Imaging changes in osteosarcoma usually have patchy calcifications. The classic imaging changes are called Codman triangle and sunburst appearance (8).

The treatment of osteosarcoma is based on surgical treatment and supplemented by chemotherapy. Surgical treatment requires complete resection of the patient ‘s tumor site (amputation or limb salvage surgery) (9). Surgeons should record the size of the tumor in the resected bone at the time of the resection of the primary tumor and judge whether it is thorough according to pathological section (10). Chemotherapy includes preoperative chemotherapy and postoperative chemotherapy, but the effect of postoperative chemotherapy is not satisfactory, and whether postoperative chemotherapy can significantly prolong the survival period of patients remains questionable (11). Some of the most effective drugs for treating osteosarcoma include cidplatin, doxorubicin, methotrexate, and ifosfamide (see **Table 1**). The effect of radiation therapy is generally considered negligible for osteosarcoma (12). The standard treatment is to use neoadjuvant chemotherapy (chemotherapy before surgery) followed by surgical resection (9). The prognosis of osteosarcoma is usually dismal and important for patients to keep lifelong follow-up monitoring because distant metastatic osteosarcoma usually occurs within about a decade of diagnosis (13, 14).

**Table 1:** NCCN Guidelines Version 2.2023 about Osteosarcoma (www.nccn.org/patients).

	Preferred regimens	Other recommended regimens	
First-line therapy (primary/neoadjuvant/adjuvant therapy or metastatic disease)	• Cisplatin and doxorubicin • MAP (high-dose methotrexate, cisplatin, and doxorubicin)	•Doxorubicin, cisplatin, ifosfamide, and high-dose methotrexate	
Second-line therapy (relapsed/refractory or metastatic disease)	• Ifosfamide (high dose) ± etoposide • Regorafenib • Sorafenib	• Cabozantinib • Cyclophosphamide and topotecan • Docetaxel and gemcitabine • Gemcitabine • Sorafenib + everolimus	Useful in Certain Circumstances • Cyclophosphamide and etoposide • Ifosfamide, carboplatin, and etoposide • High-dose methotrexate • High-dose methotrexate, etoposide, and ifosfamide • Sm^153-^EDTMP for relapsed or refractory disease beyond second-line therapy

## 2 Ubiquitin

### 2.1 Ubiquitination

**Figure 1:**
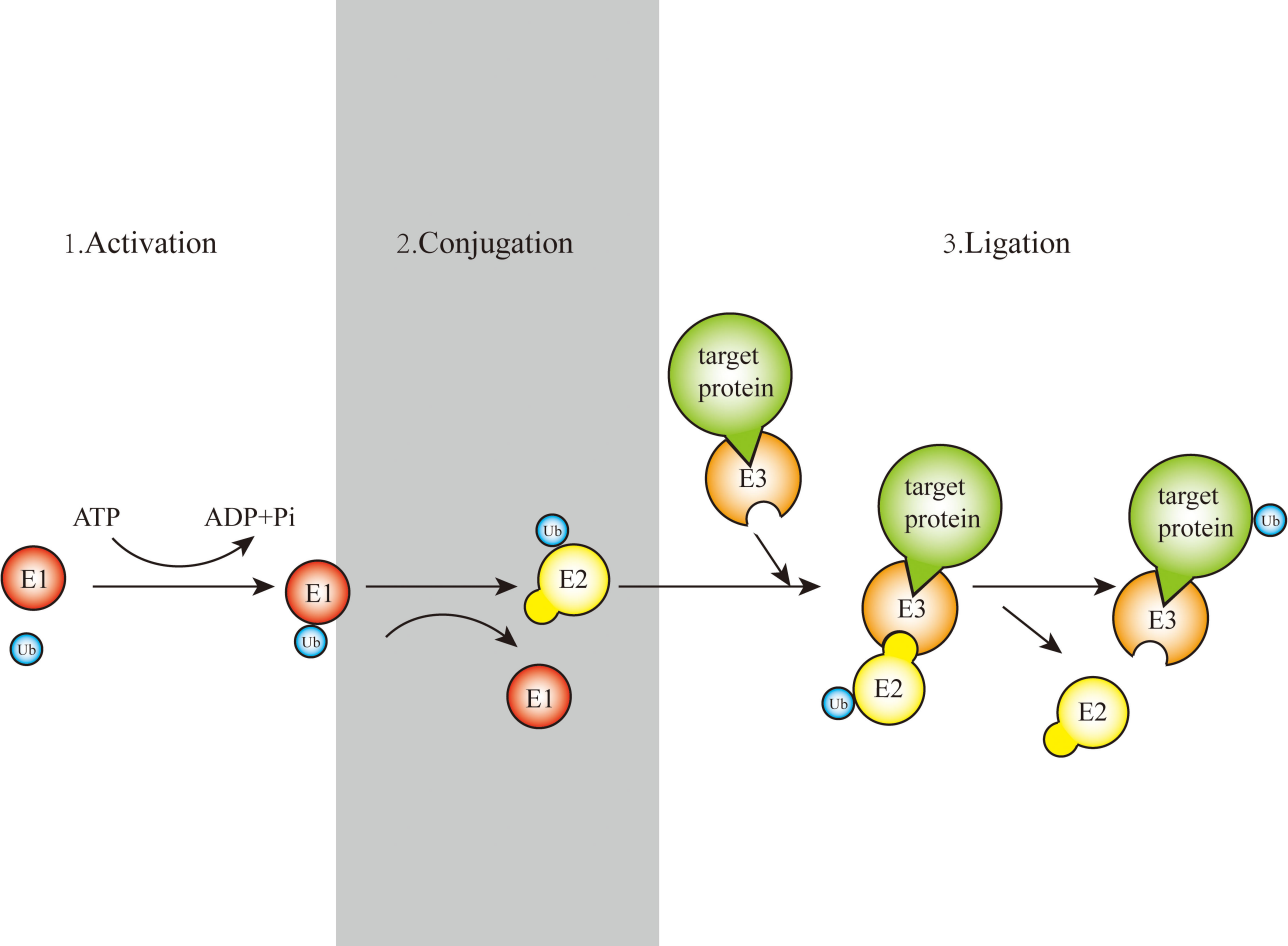
Schematic representation of the ubiquitination process.

**Figure 2:**
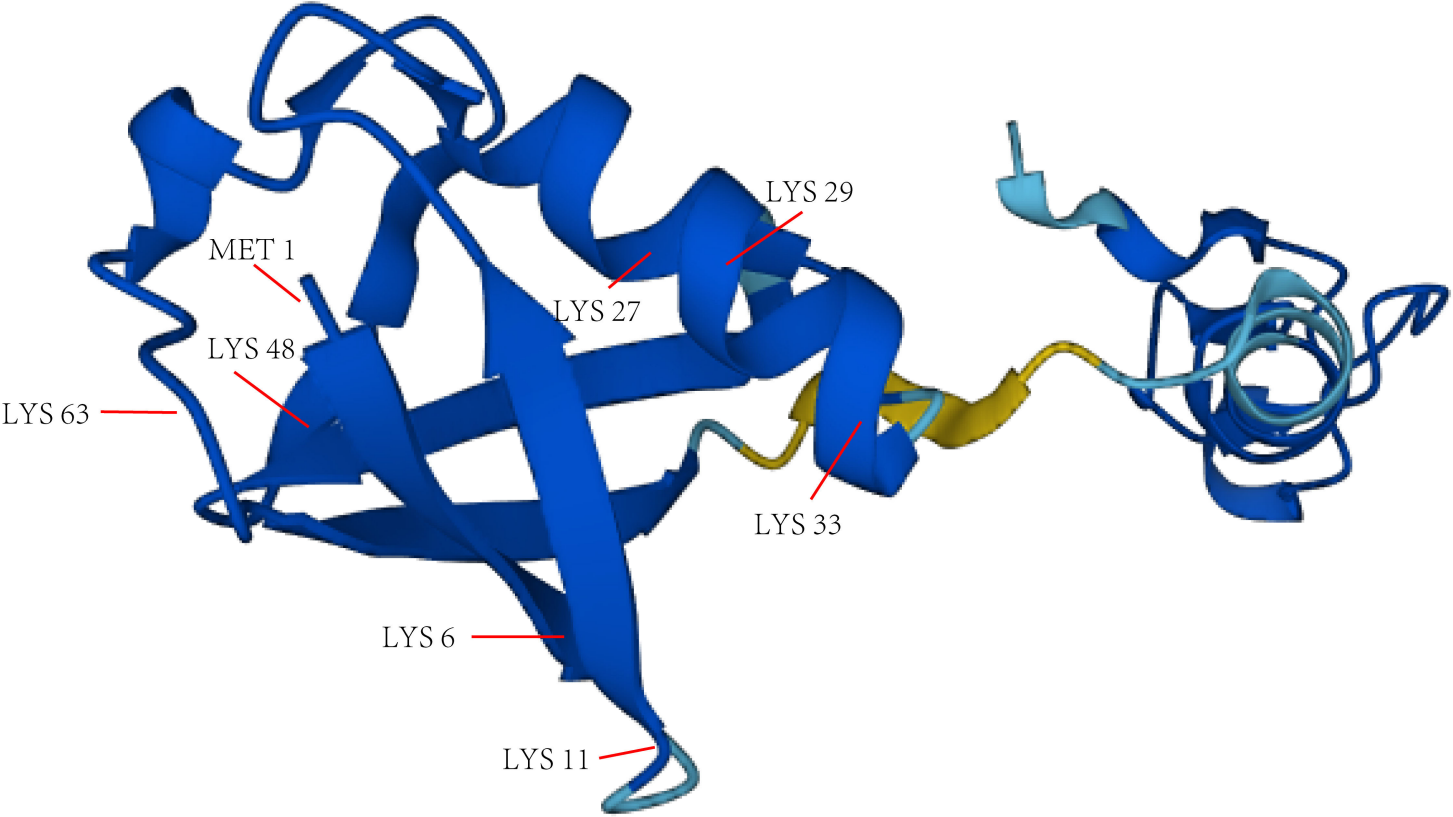
The main lysine residues and N-terminal methionine of Ub. The structure picture of Ub is from alphafold.ebi.ac.uk.

Gideon Goldstein first discovered 74 highly conserved amino acid sequences in cattle and humans since 1975. Gideon Goldstein defined them as ubiquitin (Ub) (15). As delving into study of ubiquitination, key roles for the ubiquitination has been reported in some cancers such as lung cancer, pancreatic cancer and so on (16, 17). Jin et al. summarized the role of ubiquitination in lung cancer such as tumor initiation, metabolism and survival (16). Chen et al. found that UBR5, an E3 ubiquitin ligase, was significantly upregulated in pancreatic cancer. UBR5-induced aerobic glycolysis is dependent on the ubiquitination of fructose-1,6-bisphosphatase (FBP1) in pancreatic cancer cells. These results provide the role of UBR5 in pancreatic cancer cell adaptation to metabolic stresses (17). Ubiquitin, a regulatory protein of 8.6 kDa, is widely distributed in eukaryotes (15). UBB, UBC, UBA52, and RPS27A are recognized as the genes that encode ubiquitin in the human body (18). Ubiquitination refers to the process by which ubiquitin molecules recognize and specifically modify intracellular target proteins under a cascade of the actions of ubiquitin-related enzymes. This process is accomplished by ubiquitin-activating enzymes (E1s), ubiquitin-conjugating enzymes (E2s), and ubiquitin ligases (E3s). The specific process is characterized by the first activation of Ub by E1 ubiquitin-activating enzyme, and Mg^2+^ -ATP of E1 ubiquitin-activating enzyme catalyzes the formation of covalent thioesters between cysteine on E1 ubiquitin-activating enzyme and the diglycine sequence at the C-terminus in Ub. Afterwards, E1 transfers Ub to the catalytic cysteine of E2 *via* a diglycine motif, forming an E2-Ub thioester complex. Then E3 binds the substrate with the E2- Ub complex. The ϵ-amino group or free N-terminal amino group of the lysine side chain of the C-terminal carboxy-linked substrate of Ub forms peptide bonds (see **Figure 1**). Successive rounds of E3 catalytic reactions can produce substrates with polyubiquitin chains that achieve labeling of the substrate through the seven lysine residues of Ub (Lys6, Lys11, Lys27, Lys29, Lys33, Lys48, and Lys63) or the N-terminal methionine of Ub (see **Figure 2**). Different lysine residues or methionine activate different downstream signals. For example, Lys48 activates the 26S proteasome to degrade the labeled substrate and Lys63 guides the substrate to the endocytic pathway and regulates kinase activation in the NF-κB pathway (19, 20). Recent studies reveal that K63-linked ubiquitination of IKKγ (also known as NEMO) is also critical for IκB kinase (IKK) activation. Degradation of IκB releases NF-κB from heterdimerizing with IκB and facilitates NF-κB nuclear translocation and activation (21). The family members of E1s and E2s are relatively simple. E1s are UBA1, UBA4, UBA5, UBA6, UBA7, ATG7 (22). 40 members have been described and found to be the members of E2s family. All E2s contain a highly conserved domain, termed the UBC domain, and some of the E2s have an additional N- and/or C- terminal domain. Therefore, it has been divided into four major classes. **Table 2** shows E2s with their specific domain. With nearly 600 members, the E3 ubiquitin ligases family is the most studied today, specifically divided into three families: HECT, RING-finger, and RBR domain (23–26).

**Table 2:** E2 enzymes and their specific domain.

Name	Synonyms	Domain
UBE2A	UBC2, HR6A, HHR6A, RAD6A	UBC
UBE2B	UBC2, HR6B, HHR6B, RAD6B, E2-17K	UBC
UBE2C	UBCH10, DJ447F3.2, EC 6.3.2.19	Ext-UBC
UBE2D1	SFT, UBCH5, UBC4/5, UBCH5A	UBC
UBE2D2	UBCH5B	UBC
UBE2D3	UBCH5C	UBC
UBE2D4	UBCH5D	UBC
UBE2E1	UBCH6	Ext-UBC
UBE2E2	UBCH8	Ext-UBC
UBE2E3	UBCH9	Ext-UBC
UBE2F	NCE2	Ext-UBC
UBE2G1	UBC7	UBC-insert
UBE2G2	UBC7	UBC-insert
UBE2H	UBC8	UBC- Ext
UBE2J1	UBC6	UBC-insert-Ext
UBE2J2	UBC6	UBC-insert-Ext
UBE2K	UBC1	UBC-UBA
UBE2L3	E2-F1, UBCH7, UBCM4	UBC
UBE2N	UBCH-BEN, UBC13	UBC
UBE2O	E2-230K, FLJ12878, KIAA1734	Ext-UBC-Ext
UBE2Q1		Ext-UBC-insert
UBE2Q2		Ext-UBC-insert
UBE2QL		UBC-insert
UBE2R1	CDC34, UBCH3, UBC3, E2-CDC34	UBC-insert-Ext
UBE2R2	UBC3B	UBC-insert-Ext
UBE2S	E2-EPF	UBC- Ext
UBE2T	PIG50, HSPC150, FANCT	UBC- Ext
UBE2U		UBC- Ext
UBE2V1	UEV1	Ext-UBC
UBE2V2	UEV2	UBC
UBE2W	UBC16	UBC
UBE2Z	HOYS7, FLJ13855, USE1	Ext-UBC-Ext

Ubiquitination is widely involved in intracellular substance transport, autophagy, DNA repair, and protein-protein interactions (27, 28). It is generally accepted that there are two types of ubiquitination, monoubiquitination and polyubiquitylation. The monoubiquitination process involves adding a ubiquitin protein to a substrate protein (29, 30), while polyubiquitination is the process of covalent bonding between multiple ubiquitin molecules and different amino acid residues (29, 31), and protein monoubiquitination can have an effect on the polyubiquitination of this protein (29). The HECT-type E3 ligase NEDD4-1 promotes monoubiquitination and polyubiquitylation of PTEN, which allows PTEN to enter the nucleus to prevent its degradation, while polyubiquitylation leads to PTEN degradation in the cytoplasm (32). The mechanisms that determine whether proteins undergo monoubiquitination or polyubiquitylation are unknown. There are currently two views, one that suggests that compatibility between amino acid residues in the E2 catalytic region and those in the substrate an Ub together determine ubiquitin type (33). For example, the E2-E3 complex Cdc34-SCF Cdc4 in yeast is more affinity for K53 residues for six residues at the N-terminus in substrate Sic1. Substitution of amino acid residues around K53 residues for poorly affinity K32 or K84 residues, on the other hand, decreases the affinity of Cdc34-SCF Cdc4 for K53 residues. Whereas mutants in the E2 catalytic center Cdc34 similarly alter the affinity for the K53 residue. These factors together influence whether ubiquitin complexes monoubiquitinate or polyubiquitinate substrates (34). Another view is that monoubiquitination leads to structural changes in the substrate that limit polyubiquitylation of the substrate. For example, the RING family E3 ligase Parkin blocks other ubiquitin-binding domains of Eps15 following its monoubiquitination, thereby limiting elongation of ubiquitin chain (35).

### 2.2 Deubiquitination

Deubiquitylation is usually mediated by deubiquitinase and is roughly characterized by the separation of ubiquitin from ubiquitinated proteins in the presence of deubiquitinase, and in turn reverses ubiquitination (36). For example, A20 causes K48-linked proteasomal degradation instead of K63-linked polyubiquitination of receptor-interacting serine-threonine kinase 1 (RIPK1) (37). More than 100 deubiquitinases have been identified and can be divided into seven subcategories, and the largest of which is ubiquitin-specific proteases (USP). Others are the ubiquitin C-terminal hydrolases (UCHs), the ovarian tumor proteases (OTUs), the Machado-Josephin domain proteases (MJDs), the JAB1/MPN+/MOV34 (JAMM) domain proteases (JAMMs), the monocyte chemotactic protein-induced proteins (MCPIPs), and the novel motif interacting with ubiquitin-containing DUB family (MINDY) (36, 38). Recently, some scholars have proposed new subclasses including Zn-finger and UFSP domain proteins (ZUFSPs) (36).

### 2.3 The function of ubiquitination/deubiquitination in cancer

Current evidence suggests that ubiquitination/deubiquitination impacts cancer development in a number of ways. The TGF-β pathway is considered to be an important signal pathway in the development process of cancer, preventing cancer from development by inhibiting cell proliferation and promoting apoptosis in the early stage, and promoting the growth of tumors by stimulating epithelial-mesenchymal transition, distant metastasis of tumors, and evading the immune system in the late stage (39, 40). USP4 and USP15 have been shown to play an important role in the process of tumor metastasis. USP4 regulates breast cancer metastasis through the Relaxin/TGF-β1/Smad2/MMP-9 pathway (41). USP15 can bind SMURF2 to deubiquitinate the TGF-β type I receptor and thus affect tumor development (42). NF-κB pathway is generally thought to suppress apoptosis and promote inflammatory responses which significantly regulate the development of cancer (43, 44). As an important deubiquitinating enzyme, A20 regulates the NF-κB pathway through the OUT domain and zinc finger domain (45). In addition, deubiquitinating enzymes such as USP4 and USP21 can also regulate the NF-κB pathway (46, 47). As a well-known tumor suppressor gene that regulate cell cycle and apoptosis, P53 deletion leads to enhanced glycolysis and maintenance of redox homeostasis in tumor cells (48). The MDM2/MDMX complex is an important E3 ubiquitin ligase and negative regulator of P53 which reduces P53 expression and promotes degradation of P53 during tumor development (49). USP7 is thought to inhibit the ubiquitination process of MDM2 and MDM4, and inhibition of USP7 expression induces apoptosis and leads to cell cycle arrest in tumors (50, 51). Therefore, USP7 may be a useful therapeutic target. In addition, several other deubiquitinating enzymes like USP2a, USP4, USP5, USP9X, USP10, USP11, USP15, USP24, USP29, and USP49 have also been shown to be associated with P53 (52). Moreover ubiquitination/deubiquitination also regulates tumor development in some other pathways. Previous study has indicated that USP9X can regulate tumor development through the Wnt signaling pathway by regulating the expression of DVL2 (53). USP7 can then affect hepatocarcinogenesis by regulating the PI3K-AKT pathway (54).

## 3 Ubiquitination/deubiquitination in osteosarcoma

### 3.1 E2 ubiquitin conjugating enzymes

E2 ubiquitin-conjugating enzymes have not been extensively studied in osteosarcoma. Chen et al. found that the E2 ubiquitin-conjugating enzyme UBE2O decreased Bmal1 expression, and UBE2O knockdown enhanced the amplitude of the U2OS circadian clock in osteosarcoma cells (55). UBE2T, a member of the E2 ubiquitin-conjugating enzyme family, was significantly highly expressed in osteosarcoma. Wang et al. showed that UBE2T knockdown could inhibit the PI3K/Akt signaling pathway to exert its inhibitory effect on osteosarcoma development (56).

### 3.2 E3 ubiquitin ligases

#### 3.2.1 RING E3 ubiquitin ligases

In previous studies, it was found that two proteins in the MDM family, MDM2 and MDMX, can either individually regulate the P53 gene or synergistically inhibit the expression of the P53 gene (57). The Ring domain of MDM2 exhibits E3 ubiquitin ligase activity, whereas the Ring domain of MDMX does not (58). As a result of their interactions with P53, both proteins are known to influence osteosarcoma development (58). Long noncoding RNAs (lncRNAs) have been shown to play critical regulatory roles in the proliferation, differentiation, and apoptosis of osteosarcoma cells (59). Guan et al. found that lncRNA PCAT6 promoted proliferation, migration, and invasion of osteosarcoma by increasing MDM2 expression (60).

As a member of the E3 ubiquitin ligase family, the Skp1-cullin-F-box (SCF) complex is able to participate in both substrate recognition, ubiquitination recruitment, and degradation in the ubiquitin proteasome system. F-box family members belong to a critical subunit of the SCF complex, and F-box39 is aberrantly expressed in many tumors (61), Inhibition of F-box39 expression in U2OS cells has been found to inhibit tumor metastasis and promote tumor cell apoptosis (62).

TRIM family members are E3 ubiquitin ligases whose structure contains a RING loop, one or two B-Box and coiled-coil (RBCC). Among them, the RING finger region has E3 ubiquitin ligase activity and can specifically bind to E2 ubiquitin conjugating enzyme in order to regulate different substrates (63). The TRIM family has been extensively studied in osteosarcoma. Wang et al. demonstrated that TRIM11 expression was significantly upregulated in osteosarcoma cells. TRIM11 was able to ubiquitinate DUSP6, regulate the ERK1/2 pathway and promote osteosarcoma growth (64). Jiang et al. demonstrated that TRIM46 expression was upregulated in osteosarcoma by interacting with PPARα and promoting its ubiquitination. Meanwhile, TRIM46 regulated the NF-κB signaling pathway to promote osteosarcoma cell growth and inhibit their apoptosis (65). Yuan et al. found that TRIM58 expression was markedly downregulated in osteosarcoma cells and co-acted with PKM2 to inhibit glucose consumption and lactate secretion in order to inhibit osteosarcoma development (66). Zhou et al. found that TRIM7 expression was up-regulated in osteosarcoma and could also bind to BRMS1 and promote its ubiquitination, resulting in enhanced migration and invasion of osteosarcoma cells and drug resistance activity, especially against MTX (67).

CRL (Cullin-RING E3 ubiquitin ligase) is the largest E3 ligase family in eukaryotes, and the human cullin family is mainly composed of eight closely related proteins (CUL1, CUL2, CUL3, CUL4A, CUL4B, CUL5, CUL7, and CUL9) (68). CUL4 usually complexes with E3 ubiquitin ligases composed of RBX1, DDB1, and DCAF, and CRL4 all share a similar core structure, with E3 ligase activity determined by CUL4-RBX1 and substrate specificity controlled by DCAF, and the CUL4 subfamily includes two members, CUL4A and CUL4B (69). Chen et al. demonstrated that CRL4B expression was overexpressed in osteosarcoma and could ubiquitinate p21. CRL4B knockdown can arrest the osteosarcoma cell cycle in S phase and attenuate cell proliferation (70). TNF as a well-known inflammatory cytokine can induce activation of the NF-κB pathway (71). Activation of the TNF-α/NF-κB axis enhanced E3 ubiquitin ligase CRL4B^DCAF11^ activity and modulated cell cycle progression in human osteosarcoma cells (72). Recent studies have identified a subset of microRNAs (miRNAs/miRs) that regulate osteosarcoma development and may serve as molecular drug targets (73, 74). MiR-300 regulates PTEN ubiquitination through the E3 ligase CRL4B^DCAF13^ to influence osteosarcoma development (75). As an artificially selected biological small molecule, TSC01131 showed toxicity against tumor cells and inhibited the growth of yeast cells and osteosarcoma cells (75). Chen et al. showed that TSC01131 inhibited osteosarcoma cell growth by reducing substrate ubiquitination by the E3 ubiquitinase CRL4B (76).

In addition to the ubiquitin enzymes in these families regulating the process of osteosarcoma, some ubiquitin enzymes are still closely related to osteosarcoma progression. RLIM, encoding an E3 ligase, has been found to ubiquitinate STMN1 in osteosarcoma in order to decrease STMN1 expression. However, compared with normal cells, G2/M phase processes were significantly increased in MG-63 cells overexpressing RLIM and Saos-2 cells (77). Chen et al. found that the E3 ubiquitin ligase SPOP was down-regulated in osteosarcoma and could inhibit osteosarcoma invasion through the PI3K/AKT/NF-κB signaling pathway (78). The E3 ubiquitin ligase c-Cbl can inhibit tumor cell growth by inhibiting targeted receptor tyrosine kinases (RTKs) and inhibit lung metastasis of osteosarcoma (79). DTX1, as a RING domain-containing E3 ubiquitin ligase, is able to cooperate with HES1 to regulate NOTCH signaling pathway to inhibit osteosarcoma invasion (80). Li et al. found that the E3 ligase TRAIP was highly expressed in osteosarcoma and could polyubiquitinate KANK1 to activate the IGFBP3/AKT pathway and promote proliferation of osteosarcoma cells (81). Geranylgeranylacetone (GGA) as an oral antiulcer agent that acts as an inducer of heat shock protein 70 (Hsp70) (82), has been shown to promote apoptosis in human osteosarcoma cells by inducing PRMT1 degradation *via* the E3 ubiquitin ligase CHIP (83). Cdc20 is generally considered an activator of the E3 ubiquitin ligase APC/C (84). Studies have demonstrated that acpin (85), an inhibitor of Cdc20, is thought to inhibit proliferation and metastasis of osteosarcoma by inhibiting APC/C activation by Cdc20 (86).

#### 3.2.2 HECT E3 ubiquitin ligases

SMURF1 and SMURF2 are HECT-type E3 ubiquitin ligases, which have been found to regulate SMAD family protein stability in the TGF-β/BMP signaling pathway (87). Uev1A is a member of the UEV family of E2 ubiquitin-conjugating enzyme variants, which lack active Cys residues with ubiquitination (88). Zhang et al. found that Uev1A could promote smad1 ubiquitination by SMURF1 to promote cell differentiation of osteosarcoma (89). Huang et al. found that SMURF2 bound the E3 ubiquitin ligase RLIM and regulated the TGF-β pathway to promote metastasis in osteosarcoma (90).

HACE1, a HECT E3s family member (91), reduces ROS levels *in vitro* and *in vivo* by blocking NADPH oxidase-mediated superoxide generation (92). It has been found that the HACE1 expression is decreased in osteosarcoma and inhibits tumor development by ubiquitinating RAC1 (93).

However, we find that there are few studies of RBR E3 ubiquitin ligase in osteosarcoma.

### 3.3 Deubiquitinylating enzymes

Numerous studies have confirmed that deubiquitinating enzymes are widely involved in the development of osteosarcoma. Previous studies have demonstrated that DNA-binding inhibitors (IDs) antagonize basic-helix-loop-helix (bHLH) transcription factors to inhibit cell differentiation and keep maintenance of stem cell status (94, 95). Williams et al. found that USP1 expression was overexpressed in osteosarcoma and USP1 could bind and deubiquitinates ID1, ID2, and ID3. Moreover, USP1 inhibits bHLH-dependent expression of CDKI p21, inhibits osteoblast differentiation, and leads to uncontrolled proliferation of osteosarcoma cells (96, 97). In addition, it has been found that down-regulation of USP1 in osteosarcoma cells inhibits the expression of a variety of genes including SIK2, MMP-2, GSK-3β, Bcl-2, STAT3, cyclin E1, Notch1, Wnt-1 and cyclin A1. Moreover, inhibition of USP1 expression suppresses tumor growth in osteosarcoma (98). miR-192-5p has been identified to suppress osteosarcoma initiation and progression by targeting USP1 (99).

Through statistical studies, Lavaud et al. found that patients with highly expressed USP6 and USP41 had significantly decreased survival (100). BRCA1-associated protein-1 (BAP1), an important nucleus-associated deubiquitinating enzyme, was significantly decreased in osteosarcoma, and was able to affect metastasis and invasion of osteosarcoma cells by regulating the PI3K/AKT pathway (101). Zhang et al. demonstrated that USP22 was highly expressed and acted as a pro-oncogene *via* PI3K/Akt pathway in osteosarcoma (102). Zeng et al. found that USP7 was significantly upregulated in osteosarcoma and could directly bind to β-catenin and activate the Wnt/β-catenin signaling pathway in order to induce epithelial-mesenchymal transition (EMT) (103). Gan et al. demonstrated that USP39 expression was overexpressed in osteosarcoma and that USP39 knockdown arrested osteosarcoma cells in G2/M phase, thereby inhibiting tumor growth and metastasis (104). MiR-140 suppresses osteosarcoma progression by inhibiting USP22-mediated LSD1 stability, resulting in promoting ubiquitination of LSD1 and increasing p21 expression (105). lncRNA DSCAM-AS1 has been demonstrated to increase USP47 expression through sponging miR-101-3p to promote osteosarcoma progression (106).

### 3.4 Ubiquitination altered by other genes

In some osteosarcoma related studies, although the relevant ubiquitinase has not been well clarified, ubiquitination is considered to be involved in tumor progression. Previous studies have demonstrated that DCB1 can synergize with androgen receptor (AR) and is concomitantly expressed in malignancies (107, 108). Wagle et al. showed that DCB1 knockdown enhanced AR ubiquitination and degradation, which in turn inhibited proliferation and invasion of osteosarcoma cells (109). All-trans retinoic acid (ATRA) induced osteoblastic differentiation of osteosarcoma cells both *in vivo* and *in vitro (* 110). Zhang et al. found that E2F1 specifically bound with RARα and promoted its ubiquitination leading to RARα degradation, which in turn disrupted the function of ATRA-induced osteogenic differentiation (111). lncRNA EPIC1 inhibits viability and invasion of osteosarcoma cells by promoting ubiquitination (112). Overexpression of MEF2D is associated with progression of bone malignancies (113). FAM83H can influence osteosarcoma progression by regulating β-catenin expression and ubiquitinating β-catenin (114). NRF2 has been recognized as a central hub for neutralizing ROS and restoring cellular redox balance (115), and pu et al. found that lncRNA-LAMTOR5-AS1 inhibited the ubiquitination degradation of NRF2 and thereby inhibited osteosarcoma cell proliferation and multidrug resistance in osteosarcoma (116).

## 4 Conclusions and prospects

At present, the important difficulty in the treatment of osteosarcoma lies in diagnosis, integrated strategy for inhibiting the distant metastasis, and improving the sensitivity of tumors to chemotherapeutic drugs. Increasing studies have demonstrated a relationship between ubiquitination and osteosarcoma progression (see **Table 3**). Ubiquitination process usually affects the invasion and migration, cell resistance, cell growth and other aspects to regulate the development of tumors. Thus, the studies of ubiquitination have a very positive significance for treatment of osteosarcoma. Moreover, some ubiquitin enzyme families and deubiquitinase families are widely involved in the regulation of osteosarcoma, such as, TRIM family and USP subgroup. Whether these ubiquitin enzyme families will become targets for the treatment of osteosarcoma remains further exploration. In addition, no-coding RNAs, such as miRNAs and lncRNAs, can also affect the progression of tumors by affecting the expression of ubiquitinase. They also reveal the link between osteosarcoma and ubiquitination from a more microscopic perspective.

**Table 3:** Osteosarcoma related ubiquitin enzymes and deubiquitinylating enzymes.

Types	Ubiquitin/deubiquitinylating enzymes		
E2 ubiquitin conjugating enzymes	UBE2O(55), UBE2T(56)		
E3 ubiquitin ligases	RING E3 ubiquitin ligases	MDM family	MDM2(58, 60)
		Skp1-cullin-F-box (SCF)(62)	
		TRIM family	TRIM11(64), TRIM46(65), TRIM58(66), TRIM7(67)
		Cullin-RING family	CRL4B(70, 72, 75, 77)
		Other RING E3 ubiquitin ligases	RLIM(78), SPOP(79), c-Cbl(80), DTX1(81), TRAIP(82), CHIP(84), APC/C(87)
	HECT E3 ubiquitin ligases	SMURF1(90), SMURF2(91), HACE1(94)	
Deubiquitinylating enzymes	USP1(97, 99, 100), BAP1(102), USP22(103, 106), USP7(104), USP39(105), USP47(107)		

**Figure 3:**
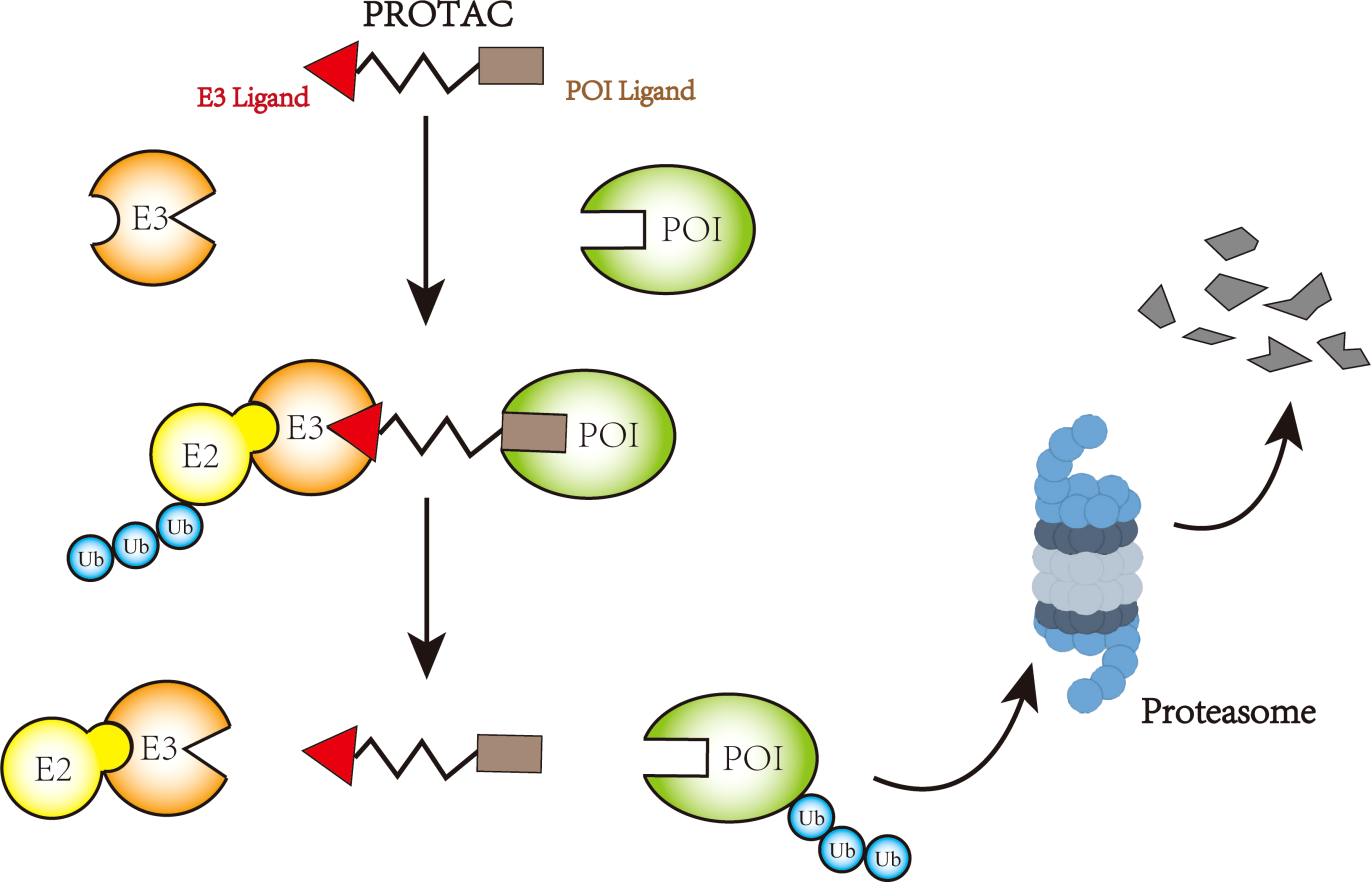
Mechanism of PROTAC-mediated protein degradation.

At present, targeted therapy is tried to replace traditional chemotherapy for cancer treatment. Targeted therapy inhibits cancer proliferation and progression by interacting with protein of interest (POI). The advantage is that it only targets specific proteins or cells and has little effect on normal tissues. However, it has been found that conventional targeted small molecule drugs probably develop drug resistance, and one of the mainstreams is mutation which cause POI to no longer strongly interact with drugs. Another resistance mechanism is that cancer can evade or become insensitive to drugs through overexpression of POI or adaptation to another signaling pathway for growth or survival. In response to these problems, attempts are being made to target proteins for degradation by endogenous mechanisms. Importantly traditional idea about protein regulation are to modify proteins through DNA or RNA. In recent years, with the maturity of CRISPR/Cas9 and RNA interference (RNAi) technology (117–119), DNA and RNA technologies have become increasingly important for protein regulation. However, both types of protein regulation are indirect, and some highly active proteins may take a long time to be completely degraded, and even resistance to degradation may occur (120, 121). At this stage, there are increasing attempts to perform endogenous protein degradation by ubiquitinase. For example, the Trim-Away system has been established to target the E3 ligase TRIM21 to proteins by using antibodies, resulting in protein degradation (122). Similar studies have also included the Shield-1 system (123). At present, the relatively mature targeted protein degradation mode is proteolytic targeted chimera (PROTAC). PROTAC is a heterobifunctional small molecule consisting of two active domains and an adaptor capable of removing specific unwanted proteins. PROTAC, first introduced by Sakamoto KM et al. in 2001 (124), consists of two covalently linked protein-binding molecules: one capable of binding to an E3 ubiquitin ligase and the other to a target protein for degradation (see **Figure 3**) (125–127). At present, some E3 ubiquitin ligases can be degraded by PROTAC technology, such as VHL (128, 129), CRBN (130, 131), MDM2 (132), β-TRCP (124), cIAP (133), RNF4 (134), RNF14 (135), and DCAF16 (136). PROTAC which consists of VHL as E3s has been studied in osteosarcoma (137). PROTAC formulations for other tumors have begun to gradually enter clinical trials, with PROTACs ARV-110 (NCT03888612) and ARV-471 (NCT04072952) entering clinical phase II trials (127). In addition, deubiquitylation techniques have also achieved some results, and cancers are believed to be driven by abnormally ubiquitinated and degraded proteins. Cancers would benefit therapeutically from targeting protein stabilization (TPS). Therefore, deubiquitinase targeting chimeras (DUBTACs), a heterobifunctional small molecule consisting of a targeting ligand and deubiquitinase recruitment group that stabilize targeted proteins degraded by abnormal ubiquitination, have been proposed. Scholars have verified the function of this molecule by experiments on the stability of the hepatocellular carcinoma inhibitory kinase WEE1 (138). During the next few years, it is expected that more and more ubiquitinases or other targeted protein degradation methods will become key treatment options for osteosarcoma.

## Data availability statement

The original contributions presented in the study are included in the article/supplementary material. Further inquiries can be directed to the corresponding author.

## Author contributions

JS, XY, reviewed the literature and wrote the manuscript. LP, JW, and PW revised the manuscript. MZ, JL, and ZL reviewed, revised the manuscript and performed figures and tables of the manuscript. All authors contributed to the article and approved the submitted version.

## Funding

This work was supported by the Youth Science and Technology Talent Program of Changzhou Health and Family Planning Commission (QN202106 and QN202007), Changzhou Sci&Tech Program (CJ20210085), and Youth Talent Development Plan of Changzhou Health Commission (CZQM2020047).

## Conflict of interest

The authors declare that the research was conducted in the absence of any commercial or financial relationships that could be construed as a potential conflict of interest.

## Publisher’s note

All claims expressed in this article are solely those of the authors and do not necessarily represent those of their affiliated organizations, or those of the publisher, the editors and the reviewers. Any product that may be evaluated in this article, or claim that may be made by its manufacturer, is not guaranteed or endorsed by the publisher.
